# The Paclitaxel Biosynthesis Pathway Unlocked

**DOI:** 10.34133/research.0965

**Published:** 2025-10-21

**Authors:** Laure Martinelli, Nicolas Papon, Vincent Courdavault

**Affiliations:** ^1^ Biomolécules et Biotechnologies Végétales, BBV, UR2106, Université de Tours, Tours, France.; ^2^ Univ. Angers, Univ. Brest, IRF, SFR ICAT, F-49000 Angers, France.

## Abstract

Paclitaxel, a natural product produced by yew trees, has become an important drug in the fight against cancer. Although more than 4 decades of research have identified numerous enzymes involved in paclitaxel synthesis, our understanding of this pathway’s architecture remains incomplete, and a few enzymes have yet to be identified. In a new article published in *Nature*, McClune and colleagues have developed a pipeline of multi-omics approaches to unravel the remaining enzymes performing key steps in the paclitaxel biosynthetic pathway. These findings open up new possibilities for producing this major anticancer drug using innovative biotechnological processes.

From a clinician’s perspective, there is a distinction in the field of oncology between before and after paclitaxel’s discovery. Paclitaxel—the molecule sold under the name Taxol—and its analogs are one of the success stories of natural product drug discovery: the combination of low toxicity and high potency led to their extensive applications in the treatment of various types of cancer [1]. As with many natural products, paclitaxel was first isolated from plants and is particularly abundant in the bark of yew trees (species from the genus *Taxus*). Since its discovery in the late 1960s, its rapidly expanded role as standard care in cancer treatments has generated a constant high demand for the compound (Fig. 1). Yet again, as with many natural products, production is complex: natural extraction is too destructive, while chemical synthesis is too expensive and not sustainable. Both chemists and molecular biologists have spent decades of research and resources understanding the synthesis of paclitaxel and other taxane molecules [1], for one, because of the tetracyclic core skeleton and, for another, because of the various hydroxyl and acetyl groups. Today, the most efficient production requires semisynthetic processes using both the bio-production of precursors and chemical synthesis [1]. One of the high-potential alternatives could be complete de novo heterologous production, which requires enzymes involved in paclitaxel biosynthesis to be known and cloned [2]. So far, a biosynthetic pathway of at least 19 steps has been proposed, and more than 15 associated enzymes have been characterized [3,4]. Obtaining a stable and sustainable production of paclitaxel in heterologous systems remains challenging and limited (Fig. 1). On one hand, the multiplicity and complexity of taxane pathways in *Taxus* species render gene identification challenging, and on the other hand, efficient production in a heterologous system often hinges on more than simply finding functional enzymes—substrate and product specificity are always to be considered. By chance, advances in structure elucidation, genomics, and other omics as well as gene transformation and expression in heterologous systems lately played a crucial role in unlocking many steps of complex biosynthetic pathways underlying important plant drug production [5], including paclitaxel [2].

**Fig. 1.**
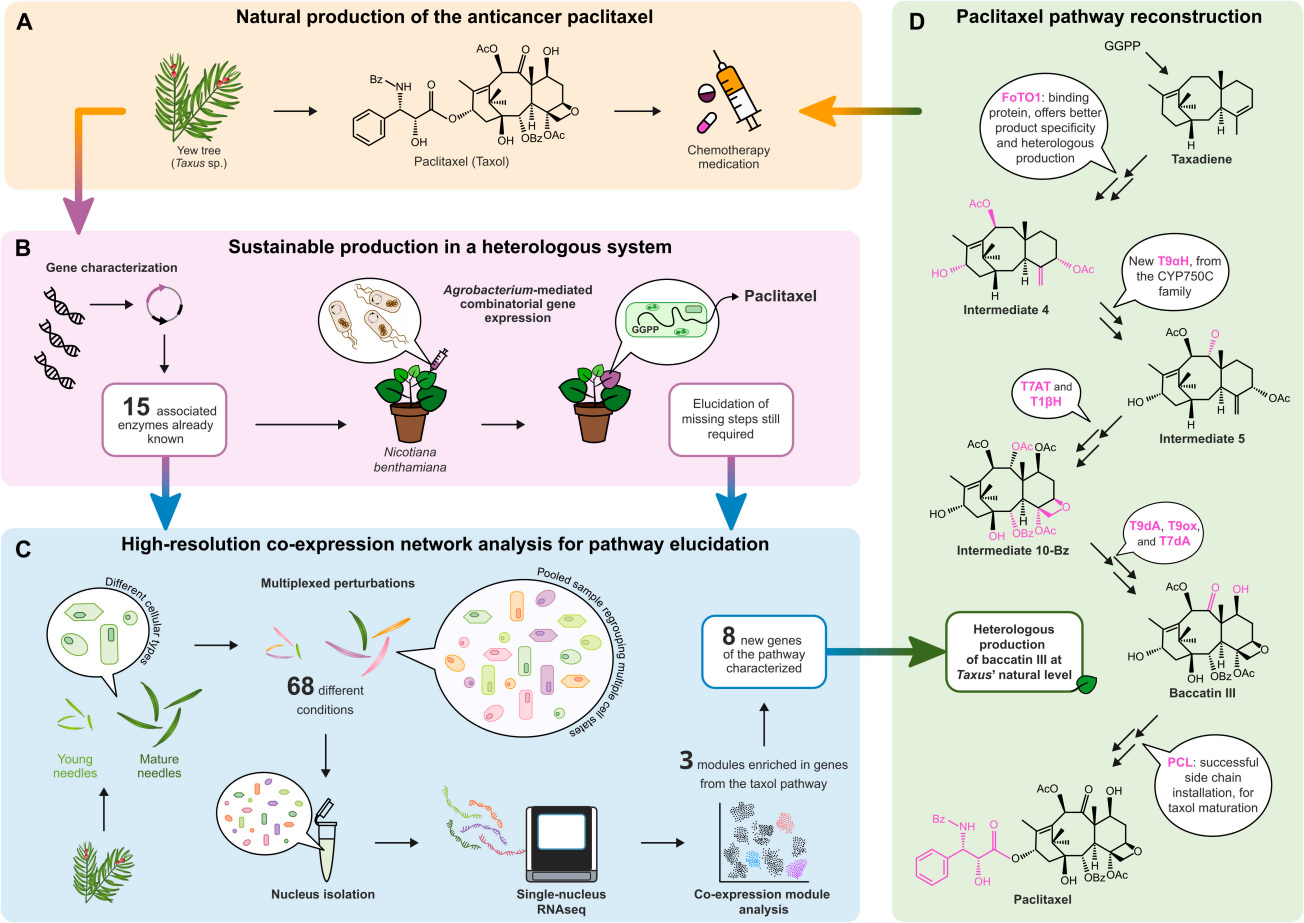
Multiplexed perturbations coupled with single-nucleus RNA sequencing (sn-RNAseq) for unlocking the heterologous production of baccatin III. (A) Paclitaxel is naturally produced by *Taxus* species, but in nonsustainable amounts, incompatible with the current Taxol clinical use. (B) Gene characterization of the paclitaxel biosynthetic pathway has been essential to achieve production of paclitaxel precursors in a heterologous system. *Agrobacterium*-mediated transient transformation of *Nicotiana benthamiana* is a promising and extensively studied alternative for sustainable production of paclitaxel. (C) Schematic overview of the methodology that allowed the characterization of 8 new genes involved in paclitaxel biosynthesis. Nucleus isolation followed by single-nucleus RNAseq was performed on a single sample, which was generated by pooling 272 needles: 68 conditions of elicitation were tested (17 elicitation treatments, each with 4 different incubation times) in duplicate and on both young and mature needles. The subsequent co-expression analysis generated 200 modules with a high enough resolution to isolate 3 modules enriched in genes from the paclitaxel biosynthetic pathway. (D) Reconstitution of the baccatin III pathway in *N. benthamiana* with 7 of the 8 new genes and previously characterized enzymes led to the production of the paclitaxel precursor at the same level as the one found in yew. Further reconstitution with additional enzymes, including the eighth newly characterized enzyme led to the successful installation of the phenylalanine-derived side chain required for paclitaxel formation. This last achievement greatly facilitates the complete reconstitution of the paclitaxel pathway in *N. benthamiana*. All of the structural differences between intermediates are highlighted in pink. Independently, the newly characterized enzymes are also highlighted in pink and positioned according to their role in the pathway. GGPP, geranylgeranyl diphosphate; FoTO1, facilitator of taxane oxidation; T9αH, taxane 9α-hydroxylase; T7AT, taxane C-7β-*O*-acyltransferase; T1βH, taxane 1β-hydroxylase; 10-Bz, 2α-*O*-benzoylated taxane intermediate; T9dA, taxane 9α-*O*-deacetylase; T9ox, taxane C-9-oxidase; T7dA, taxane 7β-*O*-deacetylase.

In a recent study published in *Nature*, McClune et al. [6] used single-nucleus RNA sequencing (sn-RNAseq) on needles from *Taxus media* to elucidate the paclitaxel biosynthetic pathway. With this recent technique, they obtained transcriptomic information for each individual needle cell and not “bulk” data from cells of the whole needle. sn-RNAseq recently emerged as a new powerful approach to tackle the elucidation of complex pathways of specialized metabolism [7]. However, this was first found to be insufficient to disentangle the complexity of taxane metabolism in *Taxus* as no cluster of expression related to the specific paclitaxel pathway could be identified. To address that limitation, the authors combined sn-RNAseq with a classical differential expression transcriptomic analysis. Nevertheless, differential expression analysis traditionally requires knowledge of the correct conditions to trigger compound accumulation (determined by metabolomics) and induction of the corresponding pathway (biosynthetic gene overexpression). Prior identification of these conditions is mandatory and can be yet particularly challenging, especially with complex organisms such as trees. The “tour de force” made by Sattely’s group was to skip this step, as they simply needed to generate as many states of expression of paclitaxel biosynthesis as possible. Therefore, they developed a tailored approach consisting of testing a multitude of cell states (referred to as “perturbations”, obtained from multiple treatments), in a single experiment, and requiring only one sample preparation and one sn-RNAseq analysis (Fig. 1). By using this strategy (so-called multiplexed perturbation × single nucleus [mpxsn]), McClune and colleagues generated 272 samples through the perturbation of *Taxus* needles with 17 different treatments, performed with 4 different incubation times and on either young or old needles. Once the samples were collected and pooled together, they went forward with the single-nucleus transcriptomics (sn-RNAseq). This mpxsn analysis generated 17,143 nuclear transcriptomes, estimated to represent 2,901 distinct cell states. To deal with the amount of data acquired and to isolate the information relevant to the studied pathway, they used a consensus nonnegative matrix factorization approach to define the modules of co-expression [8]. This led to the identification of 3 modules enriched in previously characterized genes involved in paclitaxel biosynthesis. These 3 modules were then browsed to investigate co-expressed genes—enriched in the same modules—and putatively involved in the same biosynthetic pathway.

Thanks to this approach, Sattely’s group identified a set of 8 new genes involved in the paclitaxel biosynthetic pathway and used them to produce unprecedented levels of baccatin III—a crucial precursor of paclitaxel—in the heterologous plant system *Nicotiana benthamiana* (Fig. 1). Most of these gene characterizations relied on the known steps of the paclitaxel biosynthetic pathway and the chemical structures of previously identified intermediates. In this respect, the authors searched for an enzyme that might be involved in the missing reactions within the 3 selected co-expression modules. This targeted search led to the characterization of 2 hydroxylases (taxane 9α-hydroxylase and taxane 1β-hydroxylase), 1 oxidase referred to as taxane C-9-oxidase, and 1 acyl-activating enzyme named PCL, due to its β-phenylalanine-CoA ligase activity (Fig. 1). In addition, their investigation gave an opportunity to refine several biosynthetic step orders, to discover new intermediates. For instance, the authors observed that the conversion of taxadiene into baccatin III includes 2 acetylation steps whose modifications are not found in the final paclitaxel molecule. This led to the additional characterization of 1 acyltransferase named taxane C-7β-*O*-acyltransferase and 2 deacetylases (taxane 9α-*O*-deacetylase and taxane 7β-*O*-deacetylase) (Fig. 1). Eventually, however, some characterizations were untargeted and relied entirely on the high resolution of the co-expression networks of the analysis. The best candidate from the selected modules was indeed unexpected and is a nonenzymatic nuclear transport factor 2-like protein that facilitates early 5α-hydroxylation specificity (apparently by acting as a scaffolding protein) and substantially increases heterologous titers (Fig. 1). While its precise role remains unknown, co-expressing this protein (named FoTO1, for facilitator of taxane oxidation) with the other baccatin III/Taxol biosynthetic enzymes led up to a 17-fold improvement in the production yield of the early steps, including a 10-fold increase in intermediate 4. The characterization of this protein resolved a long-standing issue for the heterologous production of paclitaxel precursors [9] and ultimately led to heterologous baccatin III production at an amount comparable with the natural abundance in *Taxus* needles.

Overall, the report by McClune et al. represents a major advance in the field of metabolic engineering of plant specialized metabolites. Above all, the global approach and the combination of omics strategies constitute a technological game changer in gene network analysis supporting specialized metabolism research. To unlock the last twists of the taxane engine, they indeed cloned and tested a total of 77 yew genes, which constitutes a reduced subset compared to a classical bulk RNAseq approach that would have required screening of thousands of candidates. Although similar multiplexing strategies have previously been developed and exploited in various research fields, most of these have been implemented for mammalian models. The large-scale perturbation methodology reported in this groundbreaking article thus constitutes a new powerful approach for gene search, especially for nonmodel organisms, as it requires no available genome. Aside from these technical innovations, findings compiled by McClune et al. corroborate the latest work from other groups [3,4,10,11], and taken together, they lay the groundwork for a complete heterologous production of paclitaxel. With only a few remaining steps—final characterization of the last 2 enzymes, yield optimization in an engineered production host, and scale-up—the establishment of sustainable, cost-effective manufacturing of paclitaxel is now within reach.
